# Effect of immunonutrition during concurrent chemoradiotherapy on acute oral mucositis in head and neck cancer patients: A prospective randomized study

**DOI:** 10.1371/journal.pone.0320145

**Published:** 2025-03-27

**Authors:** Pooriwat Muangwong, Tharatorn Tungkasamit, Vatanyu Samakgarn, Ausareeya Chumachote, Kittikun Kittidachanan, Imjai Chitapanarux

**Affiliations:** 1 Division of Radiation Oncology, Department of Radiology, Faculty of Medicine, Chiang Mai University, Chiang Mai, Thailand; 2 Department of Medical Services, Udon Thani Cancer Hospital, Ministry of Public Health, Udon Thani, Thailand; 3 Department of Medical Services, Lampang Cancer Hospital, Ministry of Public Health, Lampang, Thailand; University Hospital Zurich, Switzerland

## Abstract

**Objective:**

Immunonutrition (IN) supplements, designed to modulate immune system, may help reduce treatment-related toxicities. This study aimed to evaluate the efficacy of IN in reducing acute oral mucositis (OM) and other side effects in head and neck cancer patients undergoing concurrent chemoradiotherapy (CCRT).

**Methods:**

A multicenter, prospective, open-label randomized controlled study was conducted to assess the impact of IN on reducing OM and other side effects in head and neck cancer patients undergoing CCRT. Patients were randomized into either IN or control arms. Those in the IN arm received IN sachets starting one week before radiotherapy until treatment completion, while the control arm received no supplement. Treatment outcomes were evaluated using Common Terminology Criteria for Adverse Events (CTCAE) version 5 and patient-reported outcomes via National Cancer Institute’s Patient-Reported Outcomes of CTCAE (NCI-PRO-CTCAE). Mixed-effects logistic regression was performed to analyze outcomes between the groups, adjusting for chemotherapy regimens, while Chi-squared tests were used for group comparisons at specific timepoints.

**Results:**

A total of 87 patients were analyzed with 46 in IN arm and 41 in control arm. Physician evaluations showed similar rates of grade 2 or higher OM, dermatitis, esophagitis, and hematologic toxicities across both arms. Patient-reported outcomes also showed no significant differences in the severity of oral mucositis or its interference with daily activities. A significant reduction of dermatitis was observed at week 3 in the IN arm (9.8% vs. 34.8%, *p* =  0.03), but this effect was not sustained in later weeks.

**Conclusion:**

IN supplementation during CCRT did not result in statistically significant reductions in acute oral mucositis and other radiation toxicities. Further research with larger sample sizes, compliance monitoring, and optimized supplementation protocols is warranted to better understand the potential benefits of IN.

## Introduction

Immunonutrition (IN) supplements contain specific elements, including certain amino acids such as arginine and glutamine, ω−3 fatty acids, and nucleotides, that are known to modulate immunity [1]. IN supplementation has shown promising outcomes in cancer care. In surgical series, perioperative IN has been associated with improved treatment outcomes. In head and neck cancer surgery and gastrointestinal cancer surgery, studies have shown that IN support reduced systemic inflammatory response syndrome, post-operative complication rate, and length of stay [2–4].

Radiotherapy is a critical component of cancer treatment. In head and neck cancer, it can be used alone or in combination with other treatments such as chemotherapy or surgery [5,6]. Despite its effectiveness, radiotherapy can inadvertently damage the surrounding organs, causing adverse effects that can negatively impact the patient’s condition [7,8]. Acute adverse effects of radiotherapy are caused by inflammation in the irradiated tissues, and commonly present clinically as oral mucositis, dermatitis, esophagitis, and pharyngitis. These symptoms can cause significant pain and swallowing difficulty, resulting in reduced nutritional intake and weight loss [9–11].

Several radiotherapy studies have reported that IN reduces hematologic toxicity, decreases inflammation, and enhances immunity, based on various measurement methods of inflammatory and immunity [8,12,13]. Machon et al. demonstrated that IN before concurrent chemoradiotherapy (CCRT) significantly reduced C-reactive protein (CRP) and α−1 acid glycoprotein. However, IN during CCRT did not result in statistically significant reduction of CRP and α−1 acid glycoprotein levels between at the start and the end of CCRT [8]. Similarly, Zheng et al. did the meta-analysis and found that IN reduced the severe toxicities (oral mucositis, diarrhea, esophagitis), and maintained weight after the treatment compared to standard nutrition support [14].

Our previous study on IN during radiation treatment for head and neck cancer patients demonstrated that IN statistically significant reduced hematologic toxicities [15]. Additionally, the result of our other trial on various types of cancer including head-and-neck cancer, esophageal cancer, and cervical cancer showed a significant lower incidence of hematologic toxicity in the IN supplement group, while non-hematologic toxicities and two-year overall survival were not different [16]. In both studies, patients received two sachets of IN daily, initiating at the start of radiotherapy and continuing until treatment completion. The administration of these 2 sachets of IN to the patients included a diary nutrition component consisting of 31.3 g of protein (6.16 g of arginine and 3.07 g of L-glutamine) along with 2.73 g of fish oil.

In this study, we initiated the IN regimen to start one week before radiotherapy and continued throughout the treatment, with the dosage increased to three sachets per day. Our hypothesis was that this intensified IN regimen could effectively reduce acute oral mucositis during radiotherapy. Additionally, we investigated the impact of IN on other acute side effects associated with head and neck cancer radiation.

## Materials and methods

### Study design and participants

A multicenter, prospective, open-label randomized controlled study was conducted in patients with locally advanced head and neck cancer who were receiving CCRT, for either definitive treatment or postoperative setting. The study was carried out at three radiation oncology centers in Thailand, including Maharaj Nakorn Chiang Mai Hospital, Udonthani Cancer Hospital, and Lampang Cancer Hospital. The eligibility criteria were non-metastatic, non-nasopharyngeal head and neck cancer patients aged 18–70 years; Eastern Cooperative Oncology Group (ECOG) performance status of 0–1; histologically confirmed squamous cell carcinoma; no active autoimmune disease(s), diabetes mellitus, renal disease, or hepatic disease; no breastfeeding or pregnancy.

This study was approved by the Research Ethics Committees at all participating centers; including Research Ethics Committee No. 1, Faculty of Medicine, Chiang Mai University (Approval No. 320/2021); Udonthani Cancer Hospital (Approval No. UDCH_COA 016/2021), and Lampang Cancer Hospital (Approval No. 87/2564). The study is registered with the Thai Clinical Trial Registration (Registration No. TCTR20210902001) and was conducted in accordance with the Declaration of Helsinki. All participants provided written informed consent prior to their enrollment in the study.

### Interventions

Patients were randomized into two arms in a 1:1 ratio into the IN arm and the control arm. Randomization was performed at the screening stage using central computer-generated block randomization, stratified by radiotherapy centers. Patients in the IN arm received IN sachets three times daily, starting one week before radiotherapy and continuing throughout the radiotherapy course. Those in the control arm did not receive the IN supplement. Each IN sachet contained 60 g powder of medical supplement, composed of 3.13 g of arginine, 1.56 g of L-glutamine, and 1.39 g of fish oil. Consequently, patients randomized into the IN arm received an additional 762.48 kcal, consisting of 46.93 g of protein (4.68 g of glutamine and 9.39 g of arginine), 94.77 g of carbohydrates, and 21.74 g of fat (4.17 g of fish oil). Both arms received nutritional consultations at every visit to ensure adequate energy intake with balanced macronutrient distribution. Patients were also allowed to receive additional oral nutrition supplements other than IN as needed. Physicians conducting toxicity assessments were blinded to the treatment arm, although it was not possible to blind patient-reported toxicity assessments.

### Overview of cancer treatments

All patients underwent radiotherapy using one of the following techniques: three-dimension conformal radiotherapy (3D-CRT)/ intensity-modulated radiotherapy (IMRT)/ volumetric-modulated arc therapy (VMAT)/ tomotherapy techniques. Concurrent platinum-based chemotherapy was administered weekly or tri-weekly during radiotherapy. The chemotherapy regimens consist of tri-weekly cisplatin at 100 mg/m^2^, weekly cisplatin at 40 mg/m², or weekly carboplatin with an area under the curve (AUC) of 2. Induction chemotherapy or adjuvant chemotherapy was permitted, with the specific regimen determined according to each institution’s practice guidelines.

### End points and outcome measurements

The primary endpoint was oral mucositis, evaluated by physicians using the Common Terminology Criteria for Adverse Events (CTCAE) version 5 [17]. Secondary endpoints included dermatitis, esophagitis, and hematologic toxicities, assessed by physicians using CTCAE version 5, as well as patient-reported outcomes for oral mucositis and dermatitis, collected using the Thai version of the National Cancer Institute’s Patient-Reported Outcomes of the Common Terminology Criteria for Adverse Events (NCI-PRO-CTCAE) [18].

Patients were evaluated weekly by physicians for the side effects, including oral mucositis, dermatitis, esophagitis, and hematologic toxicity, using CTCAE version 5. In addition, patient-reported outcomes were collected weekly using the Thai version of NCI-PRO-CTCAE, specifically assessing mouth/throat sores severity, mouth/throat sores interference with diary activity, and radiation skin reaction.

For analysis, physician-evaluated side effects were categorized as grade 2 or higher. Patient-reported outcomes from NCI-PRO-CTCAE were categorized as follows: (1) severity of mouth or throat sores: moderate, severe, and very severe; (2) mouth or throat sores interfere with usual or daily activities: somewhat, quite a bit, and very much; and (3) severity of skin burns from radiation: moderate, severe, and very severe. These categorized incidences were reported and compared between study arms throughout CCRT.

### Statistical analysis

It was hypothesized that IN supplementation would reduce the incidence of grade 2 or higher oral mucositis, assessed by physicians using CTCAE version 5, from 61% to 30.5%. To detect this difference with a power of 0.8 and a significance level of 0.05, the required sample size was calculated to be 41 participants per arm. Considering an expected dropout rate of 15%, a total of 96 patients were enrolled in the study.

Descriptive statistics were used to summarize the data. Continuous variables were summarized using median and ranges, while dichotomous variables were expressed as numbers and percentages.

To assess the effects of IN on toxicities throughout CCRT, mixed-effects logistic regression, accounting for repeated measurements, was used to calculate odds ratios (ORs) with 95% confidence intervals (CIs). Models were further adjusted for chemotherapy regimens to produce adjusted odds ratios (aORs). As part of a post-hoc analysis, the impact of IN on grade 3–4 oral mucositis was also evaluated using mixed-effects logistic regression.

Comparisons of baseline patient characteristics and toxicity outcomes between the IN and control arms at each time point during CCRT were performed using the Wilcoxon rank-sum test for continuous variables and the Chi-squared test for dichotomous variables. These analyses were performed on a per-protocol (PP) analysis basis to account for actual protocol adherence and exclude patients with significant deviations from the protocol. A p-value less than 0.05 was considered statistically significant. All analyses were performed using STATA software version 16 (StataCorp LLC, Texas, USA).

## Results

### Patient characteristics

**Fig 1:**
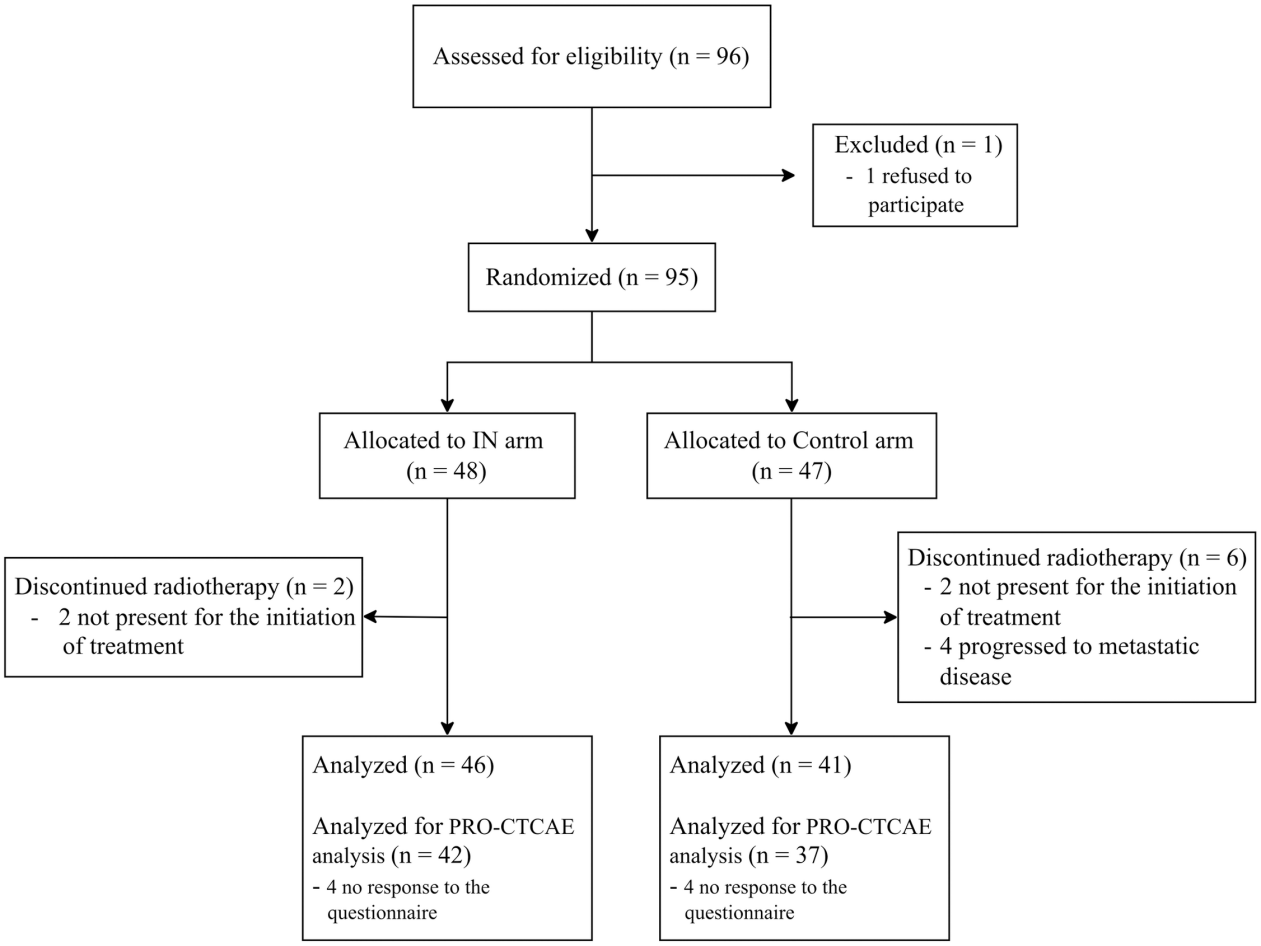
CONSORT diagram showing flow of participants.

A total of 96 patients were enrolled in the study between September 2021 and July 2023. Of these, 87 patients were included in the final analysis, consisting of 69 men and 18 women (Fig 1). The median age of patients was 55 years (range 28–70). There were 46 patients in the IN arm and 41 patients in the control arm. Patient characteristics were comparable between the two arms, except for chemotherapy regimen (Table 1). Among those who received tri-weekly cisplatin and weekly cisplatin, the median cumulative dose of cisplatin was 240 mg (range 40–300) in IN arm and 200 mg (range 40–300) in control arm (*p* =  0.04). For those who received weekly carboplatin AUC2, the median number of cycles was 6 (range 4–6) in IN arm and 6 (range 4–7) in the control arm (*p* =  0.90).

**Table 1:** Patient characteristics.

Characteristic	IN (n = 46)	Control (n = 41)
Age, year, median (range)	58 (28–70)	55 (31–69)
Sex, n (%)		
Men	34 (84.8)	35 (73.5)
Women	12 (15.2)	6 (26.5)
ECOG performance status, n (%)		
0	20 (43.5)	17 (41.5)
1	26 (56.5)	24 (58.5)
Cancer site, n (%)		
Oropharynx	21 (45.7)	15 (36.6)
Oral cavity	14 (30.4)	12 (29.3)
Hypopharynx	5 (10.9)	7 (17.1)
Larynx	4 (8.7)	6 (14.6)
Others	2 (4.4)	1 (2.4)
Cancer stage group, n (%)		
I	0 (0.0)	1 (2.4)
II	3 (6.5)	3 (7.3)
III	11 (23.9)	7 (17.1)
IVA	28 (60.9)	23 (56.1)
IVB	4 (8.7)	7 (17.1)
Treatment aim, n (%)		
Definitive	35 (76.1)	24 (58.5)
Post-operative	11 (23.9)	17 (41.5)
Radiotherapy technique, n (%)		
3D-CRT	8 (17.4)	10 (24.4)
IMRT	38 (82.6)	31 (75.6)
Chemotherapy regimen, n (%)		
Cisplain 100 mg/m^2^ every 3 weeks	3 (6.5)	10 (24.4)
Weekly cisplatin 40 mg/m^2^	36 (78.3)	22 (53.7)
Weekly carboplatin AUC 2	7 (15.2)	3 (7.3)
None	0 (0.0)	6 (14.6)
Weight, kg, median (range)	55.0 (36.9- 76.0)	54.0 (34.4–98.6)
BMI, kg/m^2^, median (range)	21.1 (14.4–30.0)	20.2 (14.5–32.0)
Serum albumin, mg/dl, median (range)	4.2 (3.5–4.9)	4.1 (2.5–5.0)

Abbreviations: IN, Immunonutrition; IQR, Interquartile range; ECOG, Eastern Cooperative Oncology Group; 3D-CRT, Three-dimension conformal radiotherapy; IMRT, Intensity-Modulated Radiation Therapy; BMI, Body Mass Index.

### Acute radiation toxicities evaluated by physicians using CTCAE

The incidence of grade 2 and higher oral mucositis steadily increased in both IN and control arms over the course of treatment. At week 3, the rates were 34.8% in the IN arm and 34.2% in the control arm. By week 4, the incidence increased to 41.3% in the IN arm and 41.5% in the control arm. At week 5, the incidence reached 58.7% in the IN arm and 51.2% in the control arm. By weeks 6 and 7, the rates in the IN arm plateaued, with 60.9% of patients reporting oral mucositis at both timepoints. In the control arm, the incidence increased slightly to 51.2% at week 6 and 61.0% at week 7.

The incidence of grade 2 or higher dermatitis remained low during the early weeks of treatment. By week 6, 28.3% of patients in the IN arm and 36.6% in the control arm reported grade 2 or higher dermatitis, increasing further by week 7 to 50.0% in the IN arm and 39.0% in the control arm. For grade 2 and higher esophagitis, the incidence was low during the first two weeks but progressively increased in both arms. By week 7, the incidence was 50.0% in the IN arm and 63.4% in the control arm.

The mixed-effects logistic regression analysis demonstrated that the incidence of grade 2 and higher acute oral mucositis as well as other radiation toxicities, as evaluated by physicians using CTCAE version 5, were comparable between the two study arms. No significant differences were observed in terms of the incidence of grade 2 and higher oral mucositis, dermatitis, esophagitis, and hematologic toxicities throughout the treatment course between the IN and control arms even after adjusting for chemotherapy regimen (Table 2).

**Table 2:** Multilevel analysis of the impact of immunonutrition on treatment-related toxicities during CCRT.

Toxicity	OR (95% CI)	p-value	aOR^*^ (95% CI)	p-value
Grade 2 and higher CTCAE				
Non-hematologic				
Oral mucositis	1.5 (0.4- 4.8)	0.54	0.9 (0.3–2.9)	0.79
Dermatitis	0.8 (0.3–2.5)	0.55	0.7 (0.2–1.7)	0.45
Esophagitis	1.0 (0.4–2.3)	0.92	0.7 (0.3–1.9)	0.54
Hematologic				
Anemia	0.5 (0.1–2.4)	0.40	0.6 (0.1–3.2)	0.55
Neutropenia	0.9 (0.4–1.8)	0.73	0.7 (0.3–1.6)	0.41
Lymphopenia	0.7 (0.3–1.8)	0.63	0.7 (0.3–1.8)	0.58
Thrombocytopenia	2.2 (0.2–20.6)	0.28	1.2 (0.1–11.1)	0.83
NCI-PRO-CTCAE				
Severity of mouth or throat sores (moderate, severe, and very severe)	0.5 (0.2–1.3)	0.15	0.5 (0.2–1.3)	0.14
Mouth or throat sores interfere with usual or daily activities (somewhat, quite a bit, and very much)	0.7 (0.3–1.6)	0.43	0.7 (0.3–1.5)	0.33
Severity of skin burns from radiation (moderate, severe, and very severe)	0.6 (0.2–1.8)	0.40	0.5 (0.2–1.4)	0.16

* Adjusted for chemotherapy regimen.

Abbreviations: OR: odd ratio; CI: confidence interval; aOR: adjusted odd ratio.

**Fig 2:**
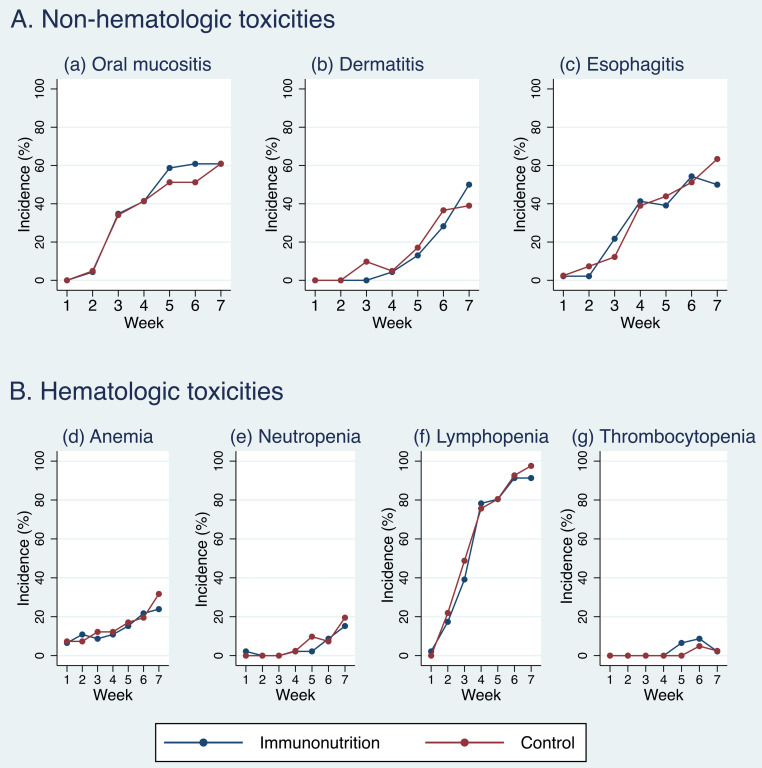
Percentage of grade 2–4 toxicities assessed by physician using CTCAE.

At each time point of CCRT, the incidence of each toxicity showed no statistically significant difference, except for dermatitis at week 3, where the IN arm had a lower incidence compared to the control arm (9.8% vs. 34.8%, *p* =  0.03) (Fig 2A).

The post-hoc analysis evaluating the incidence of grade 3 and higher acute oral mucositis using mixed-effects logistic regression revealed an OR of 0.9 (95% CI 0.2–5.4) and an aOR, accounting for chemotherapy regimens, of 1.2 (95% CI 0.2–8.1) between the IN and control arms.

The incidence of grade 2 or higher hematologic toxicities, including anemia, neutropenia, lymphopenia, and thrombocytopenia, was similar between the IN and control arms. Lymphopenia was the most common hematologic toxicity, with its incidence progressively increasing over the course of treatment. By week 7, lymphopenia had reached its highest incidence, affecting 91.3% of patients in the IN arm and 97.6% in the control arm (Fig 2B).

### Patient-reported outcomes of acute radiation toxicities

Patient-reported outcomes, assessed by NCI-PRO-CTCAE, were available for 79 patients: 42 in the IN arm and 37 in the control arm. Mixed-effects logistic regression analysis showed that patient-reported acute oral mucositis and dermatitis throughout the treatment course were comparable between the two study arms, even after adjusting for the chemotherapy regimen (Table 2).

**Fig 3:**
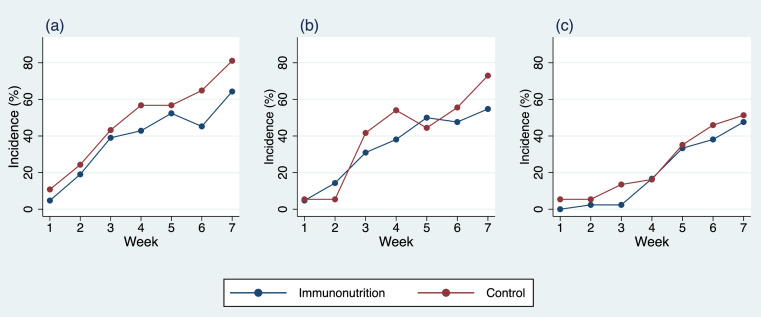
Percentage of patient-reported outcomes using NCI-PRO-CTCAE throughout radiotherapy. (a) Severity of mouth or throat sores (moderate, severe, and very severe); (b) Mouth or throat sores interfere with usual or daily activities (somewhat, quite a bit, and very much); (c) Severity of skin burns from radiation (moderate, severe, and very severe).

The severity of mouth or throat sores did not significantly differ between the IN and control arms at any time point during treatment (Fig 3). At week 6, 45.2% of patients in the IN arm and 64.9% in the control arm had moderate to severe symptoms (*p* =  0.08). At week 7, 64.3% of patients in the IN arm and 81.1% in the control arm reported the same severity (*p* =  0.08). Similarly, no significant differences were observed in the interference of mouth or throat sores with usual or daily activities at any time point, with 54.8% of patients in IN arm and 73.0% in the control arm had somewhat to very much degree of interference at week 7 (*p* =  0.09). For radiation-induced skin burns, there was no statistically significant difference between the two arms. At week 3, 13.5% of patients in the IN arm and 2.4% in the control arm reported moderate to severe skin burns (*p* =  0.06) (Fig 3).

The median overall treatment duration was 50 days (range 42–88) for the IN arm and was 50 days (range 42–85) for the control arm (*p* =  0.67). The median percentage weight change in the IN arm was −5.0% (range −15.9 to + 10.3), compared to −8.5% (range −13.4 to + 11.0) in the control arm (*p* =  0.23).

## Discussions

This study evaluated the effects of IN on acute radiation toxicities during CCRT in patients with head and neck cancer. The primary endpoint, grade 2 or higher oral mucositis as evaluated by physicians, showed no statistically significant difference between the IN and control arms throughout the treatment course after mixed-effects logistic regression analysis. A post-hoc analysis of grade 3 or higher oral mucositis similarly revealed no statistically significant differences between the arms. Other acute radiation toxicities, including dermatitis, esophagitis, and hematologic toxicities, also demonstrated no statistically significant differences between the two groups, even after adjusting for chemotherapy regimens. Patient-reported outcomes of acute toxicities were comparable between the study arms, indicating that IN did not significantly reduce the severity or interference of these toxicities.

While evaluations at individual time points revealed a significantly lower incidence of dermatitis in the IN arm at week 3, patient-reported outcomes assessed through the NCI-PRO-CTCAE indicated trends toward reductions in certain symptoms. These included a reduction in the severity of mouth or throat sores at weeks 6 and 7, reduced interference of mouth or throat sores with daily activities at week 7, and reduced severity of skin burns from radiation at week 3, which correlated with physician assessments. However, these trends did not reach statistical significance and were not sustained over the course of treatment. These findings may have limited clinical relevance, as the transient differences observed were inconsistent and not maintained throughout treatment. Additionally, the results could be influenced by repeated measurements or variability inherent in the study design.

Our findings for oral mucositis align with previous studies, including those by Boisselier et al. [19] and Dechaphunkul et al. [20], which reported that IN support during CCRT in patients with head and neck cancers did not reduce acute oral mucositis. However, other studies have shown the potential of IN to reduce oral mucositis. Assenat et al. evaluated the effect of IN in 40 head and neck cancer patients who underwent post-operative CCRT. The findings indicated that there was a trend toward a reduction of oral mucositis in patients with IN compliance more than 75% (p = 0.08) [21]. Similarly, in the study by Chitapanarux et al., although no statistical comparison was performed, grade 3 oral mucositis occurred in 1 of 20 patients (5%) in the IN arm compared to 4 of 20 patients (20%) in the control arm [15]. A meta-analysis by Zheng et al. also demonstrated that IN reduced the grade 3 or higher toxicities of oral mucositis, diarrhea, esophagitis in various cancer patients undergoing CCRT [14].

The results also showed no significant differences in the incidence of grade 2 and higher hematologic toxicities between the IN and control groups. These findings are consistent with the studies by Dechaphunkul et al. [20] and Assenat et al. [21], which reported no impact of IN on hematologic toxicities during CCRT. However, studies by Chitapanarux et al., conducted on head and neck cancer patients undergoing CCRT and on a mixed cohort of patients with head and neck cancer, esophageal cancer, and cervical cancer, reported a significantly lower incidence of hematologic toxicities in the IN arm [15,16].

Differences in outcomes may be attributed to variations in the timing, composition, and patient compliance. In our study, patients initiated IN supplementation one week before CCRT and continued daily during treatment without evaluating the compliance. This approach differs from other protocols. For example, Boisselier et al. [19], Dechaphunkul et al. [20], and Assenat et al. [21] administered IN with a different composition for five consecutive days before each tri-weekly chemotherapy cycle. Assenat et al. [21] reported a trend toward reduced oral mucositis in patients with compliance rates exceeding 75%. Moreover, the radiotherapy technique may have influenced hematologic toxicities. A retrospective review of data from our previous studies [15,16] revealed that radiotherapy techniques used were predominantly conventional and 3D-CRT, whereas the current study primarily utilized IMRT. The use of conventional radiotherapy in earlier studies likely contributed to higher rates of hematologic toxicities. These variations underscore the need for further research to determine the optimal timing, composition, duration, and compliance monitoring of IN supplementation while accounting for technological advancements in radiotherapy.

Our results integrated patient-reported outcomes and showed that oral mucositis incidence was higher compared to the oral mucositis as evaluated by physicians. A post-hoc analysis revealed that among patients with physician-assessed grade 1 and grade 2 acute oral mucositis, a considerable proportion reported moderate or higher severity of mouth or throat sore according to patient assessment (32.8% and 73.5%, respectively). This suggests potential underreporting of oral mucositis severity by physicians and suggests the potential of IN to reduce oral mucositis. Additionally, trends observed in patient-reported data, such as reduced severity of mouth or throat sores and their interference with daily activities in the IN arm during the later weeks of treatment, suggest that IN may provide benefits from the patient’s perspective. These findings warrant further evaluation in future studies with larger sample sizes to determine the significance of IN on patient-reported outcomes.

We believe that the components of IN, particularly glutamine, may have contributed to its potential for reducing oral mucositis. Glutamine is known for its protective effects on gastrointestinal mucosa integrity [22,23]. A systematic review and meta-analysis by Lyra et al. also highlighted glutamine’s role in reducing the severity and delaying the onset of mucositis [24]. Additionally, the MASCC guidelines for the management of mucositis secondary to cancer therapy also recommends oral glutamine as one of the options for oral mucositis management [25]. The effectiveness of IN may vary depending on the composition of supplements.

Our study has several strengths. First, it was a multicenter prospective randomized study, enhancing its generalizability and minimizing bias. Second, the inclusion of patient-reported outcomes provided valuable insights into the treatment effects from the patient’s perspective. Third, the weekly reporting of side effects throughout the treatment period allowed for a detailed and temporal examination of side effects.

However, the study also has limitations. First, the control arm was not blinded, potentially introducing bias into the results, particularly for patient-reported outcomes. Second, the study was conducted as a per-protocol analysis, which may limit the generalizability of the findings as it excludes non-compliant participants who could reflect real-world scenarios. Third, while patient-reported outcomes showed a trend toward reduced severity of mouth or throat sores and interference with daily activities in the IN group in the final weeks of treatment, these differences did not reach statistical significance. The relatively small sample size may have limited the balance between arms, as stratification could not be performed for every potential confounding factor, and may have been insufficient to detect smaller, clinically significant differences. Finally, the compliance of participants in the intervention group was not reported, and the level of compliance may have influenced the outcomes. Further studies using an isonitrogenic isocaloric supplement placebo-control design with larger sample, along with compliance monitoring, are needed to explore these findings.

## Conclusions

Our study showed that IN supplementation did not result in statistically significant differences in acute oral mucositis, dermatitis, esophagitis, or hematologic toxicities as assessed by physicians during CCRT. Patient-reported outcomes similarly indicated that IN did not significantly reduce the severity of oral mucositis or its impact on daily activities. These findings highlight the need for further research, including larger studies and more refined evaluation methods.

## Supporting information

S1 File Study protocol. (PDF)

S2 File Dataset. (XLSX)

S3 File CONSORT checklist. (DOCX)
