# Supplementary material for: Effect of immunonutrition during concurrent chemoradiotherapy on acute oral mucositis in head and neck cancer patients: A prospective randomized study
Source: PLoS One. 2025 Mar 27;20(3):e0320145. doi: 10.1371/journal.pone.0320145 (PMC11949368; doi:10.1371/journal.pone.0320145)
Supplement: S1 File — (PDF) [file pone.0320145.s001.pdf]

Immunonutrition Supplement for Reducing Acute Oral Mucositis in Head and Neck Cancer Radiotherapy

Literature Review

Head and neck cancer is the sixth most common cancer worldwide that accounts for more than 550,000 cases and 300,000 deaths annually. In Thailand, the incidence rate was about 110,000 per year in 2011 which was the third and the fifth most common cancer in men and women, respectively[1,2].

Radiotherapy plays an important role of head and neck cancer treatment. It is used as a single modality of treatment in early stage, and combination with chemotherapy in locally advance stage. Post-operative radiotherapy with or without chemotherapy is also be performed in head and neck cancer patient underwent surgery with adverse pathologic features.[3,4]

Even through the effective of radiotherapy and chemotherapy for head and neck cancer treatment, the side effects of each modality of treatment can cause the deteriorated effect of the patient. Both treatments also trigger pro-inflammation process[5,6].

Acute side effects of radiotherapy are including oral mucositis, esophagitis, dermatitis, decrease taste, thickening of saliva, etc. which initiate from the inflammation process of irradiated tissue. Mucositis of oral cavity, pharynx, and esophagus is resulted in the severe pain and swallowing difficulty caused the poor nutrition intake and weight loss.[7–9]

Immunonutrition (IN) is the supplement that contains elements including specific amino acids such as arginine and glutamine, ω-3 fatty acids, and nucleotides which are able to modulate the immunity[10]. Arginine and glutamine are conditionally essential amino acids especially in stress conditions. Arginine is essential element for production of nitric oxide, polyamines, proline, creatinine, and glutamate. It can increase number of T helper cell. Glutamine can protect integrity of gastrointestinal mucosa, promote immunity by being the nutrient for rapidly dividing cells such as immune cells, and is the precursor of arginine. ω-3 fatty acids such as fish oil can limit the conversion of linoleic acid into arachidonic acid and replace ω-6 fatty acids in cell membrane, therefore, it can reduce the inflammation process. Nucleotides, although have less well-defined role, are believed that they can help T cell functions.[11,12]

Supplementation of IN shows the promising outcome in cancer care. In surgical series, perioperative IN showed improvement of treatment outcomes. In head and neck cancer, and gastrointestinal cancer surgery, IN support reduced systemic inflammatory response syndrome, post-operative complication rate, and length of stay[13–15]. Base on the evidences, the European Society for Clinical Nutrition and Metabolism (ESPEN) guideline for cancer treatment recommends perioperative and postoperative IN support for cancer surgery[16] and as a part of enhanced recovery after surgery (ERAS) protocol in colorectal surgery[17].

In many radiotherapy series, IN reduced hematologic toxicity, reduced inflammation, and improved immunity based on various measurement methods of inflammatory and immunity[6,18,19]. Machon et al. demonstrated that IN before concurrent chemoradiotherapy (CCRT) significantly reduced C-reactive protein (CRP) and α-1 acid glycoprotein, but IN

during CCRT did not show statistical difference of CRP and α-1 acid glycoprotein between at the beginning of CCRT comparing with at the end of CCRT.[6] Zheng et al. did the meta-analysis and found that IN reduced the severe toxicities (oral mucositis, diarrhea, esophagitis), and maintained weight after the treatment compared to standard nutrition support[20].

Addressing the effect of IN on head and neck cancer, Chitapanarux et al. investigated a study comparing IN containing arginine, glutamine, and omega-3 fatty acid that provided daily during CCRT and no intervention in 40 head and neck cancer patients treating with CCRT. The result showed that serum albumin at the end of CCRT was higher, no significant weight loss, more completion of treatment, and less grade 3-4 hematologic toxicity in IN arm. However, there were no significant difference in the weight during the treatment and the rate oral mucositis between two groups. [21] Assenat et al. performed a phase II study evaluating effect of IN enriched with L-arginine, omega-3 fatty acid, and ribonucleotide in 40 head and neck cancer underwent CCRT. IN was prescribed 1 week before each of tri-weekly chemotherapy during CCRT. The findings indicated that there was a non-significant less mucositis in patients who complied with IN more than 75% compared to less than 75% ($p=0.082$).[22] Boisselier et al. performed a randomized control trial comparing IN which contained the same ingredient and prescribed as Assenat et al.'s study and isocaloric isonitrogenous nutrition in 180 patients. The data indicated that IN support in postoperative chemoradiotherapy in head and neck cancer didn't reduce acute oral mucositis, still, it improved long term survival[23]. Lyra et al.[24] performed a systematic review and metaanalysis and found that glutamine played a significant role on reducing severity and prolonged the onset of mucositis. The combined supplements of IN had the difference results as IN contained arginine, glutamine and omega-3 showed improved oral mucositis and treatment completion, while IN that contained arginine, omega-3 and nucleotides did not show the any significant reduction of oral mucositis.

The result of the our previous trial on various types of cancer including head-and-neck cancer, esophageal cancer, and cervical cancer showed a significant lower incidence of hematologic toxicity in the IN supplement group, while non-hematologic toxicities and two-year overall survival were not difference.[25] This was difference from the result from Boisselier et al.[23] that showed improvement of overall survival in the group with IN that had >75% compliance. We also in-house evaluated the effect of IN on the inflammation and immunity enhancement evaluated by neutrophil-to-lymphocyte ratio (NLR) and absolute lymphocyte count (ALC) using second study's data, the IN add-on failed to demonstrate a better NLR and ALC compared to control arm, however, ALC was higher at the end of radiotherapy in head and neck cancer subgroup. The result also showed the correlation of initial ALC and NLR at pre-treatment and the values during and the end of radiotherapy.

There is no recommendation on the quantities of each ingredient in combined supplement IN should be added to the regular diet and how frequent should IN be taken during the radiotherapy. Our previous studies that provided diary IN contained protein 31.3 g (arginine 6.16 g, L-glutamine 3.07 g), and fish oil 2.73 g showed that IN reduced hematologic toxicities, but no overall survival improvement.[21,25] The study result of Boisselier et al.[23] that showed a survival benefit, they provided IN that prescribed 1 week before tri-weekly chemotherapy and contained nutrition per day including protein 50.4 g (arginine 11.4 g), eicosapentaenoic acid/docosahexaenoic acid 3.9 g, ribonucleic acids 1.35 g.

With the results of our previous studies that showed the relation of pre-treatment, during and the end of treatment ALC and NLR, and the study from Boisselier et al.[23], we will study the effect of IN in head and neck cancer patients which will expand its use before radiotherapy to

modulate the immunity and inflammation before treatment and provide more intense of IN per day to reduce oral mucositis severity.

Research Hypothesis

Incidence of acute oral mucositis in head and neck cancer patients who receive IN during radiotherapy will be less compared to patients who do not receive IN.

Objective

Primary objective

1. To determine if IN can reduce cumulative acute oral mucositis of radiotherapy.

Secondary objective

1. To determine whether immunonutrition can reduce inflammation and increase immunity during radiotherapy.
2. To compare other acute side effects during radiotherapy between two arms.
3. To evaluate nutrition status during radiotherapy.

Population

Locally advanced head neck cancer patient treating with definitive or postoperative concurrent chemoradiotherapy in:

- Division of Radiation Oncology, Faculty of Medicine, Chiang Mai University.
- Radiotherapy section, Udonthani Cancer Hospital
- Radiotherapy section, Lampang Cancer Hospital

Study Design

Prospective, open-label, randomized control trial

Eligibility

Inclusion criteria

1. Age range between 18 – 70-year-old
2. Histologically confirmed squamous cell carcinoma of head and neck.
3. Locally advanced head and neck cancer planning to treat with concurrent chemoradiotherapy for definitive or postoperative setting.
4. No previous radiotherapy in head and neck area.
5. Good performance status with ECOG 0-1.
6. Laboratory tests are in the normal ranges.
7. The participant or legal representative provides the written informed consent for the study.

Exclusion criteria

1. Histological subtypes other than squamous cell carcinoma (e.g., Adenocarcinoma, small cell carcinoma).
2. Has metastatic disease or locoregional recurrence.
3. Receive immune-enhancing nutrition product prior to the randomization within 30 days.
4. Allergy to immune-enhancing nutrition ingredients used in the study.

5. Active autoimmune disease(s) that require medication within 2 years.
6. Diabetes Mellitus, renal disease, liver disease and/or other disease that limits some nutrition or calorie intake.
7. Known disease or condition that would interfere with the cooperation of the study.
8. Pregnancy or breastfeeding.

Material and Method

Recruitment and Informed Consent process

Potential volunteer who is met the inclusion criteria and not in exclusion criteria will be fully explained about the study by the investigator. The volunteer will have the sufficient time to read informed consent and have the opportunity to ask any question that may have. He/she also can ask the relative or someone he/she trusts about sign the informed consent form and is informed that the process is voluntary and can be withdrawn anytime. After fully understanding and accepting to join the study, he/she will sign the informed consent form that is approved by the local ethical committee.

Intervention Assignment

Patients will be randomized in to 2 arms:

1. IN arm
2. Control arm

Randomization

Randomization process will be done at the screening process using central randomization with computer-generated code with block randomization and stratified by radiotherapy centers. Intervention arm will be assigned randomly in 1:1 ratio.

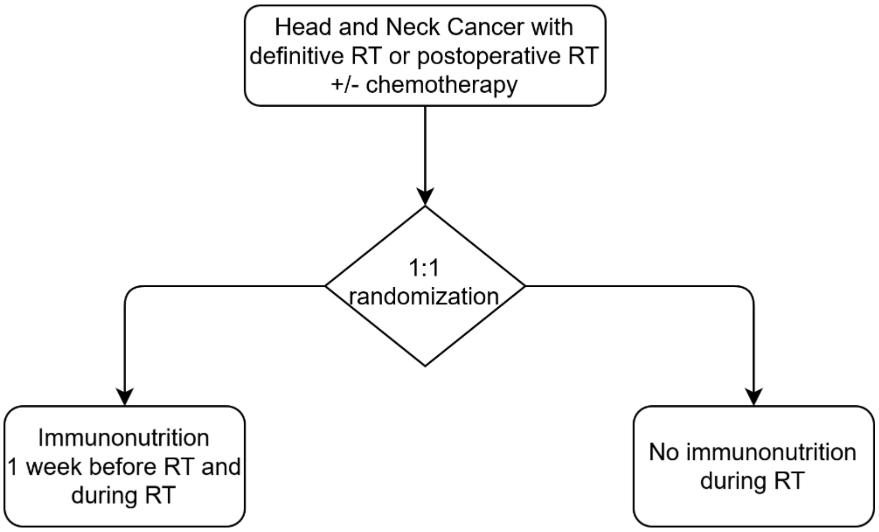

Blinding

No blinding in this study.

Nutrition support

Participant who is randomized into IN arm will receive IN 1 week before radiotherapy and daily during radiotherapy and ones in control arm will not get IN supplement. The remaining IN package will be counted in every visit.

The IN package will be provided by Thai Otsuka Pharmaceutical Co., Ltd and prepared in sachet form. Each sachet will contain 60 g powder comprising arginine 3.13 g, L-glutamine 1.56 g, and fish oil 1.39 g. It will be taken 3 sachets per day by diluted in 250 mL of water. As a result, patients who are randomized into IN arm will get 762.48 additional calories which contains 46.93 g of protein (4.68 of glutamine and 9.39 of arginine), 94.77 g of carbohydrate, and 21.74 g of fat (4.17 of fish oil).

Both arms will receive nutrition consultation every visit to ensure adequate energy intake with proper portions of macronutrients altogether with side effects of the treatment(s) and IN. Participants who experience the symptoms that limit intake such as nausea, vomiting, dysphagia, or odynophagia will be evaluated by the treating physician and receive appropriate standard treatment according to the symptom(s). Participants in IN arm are encouraged to take IN as much as they can if they have side effect(s) that limit IN intake. If patients experience a severe side effect that compromise the safety, including grade III-IV toxicity caused by IN, the IN will be withdrawn and the study will be discontinued in that particular participants.

Radiotherapy

Patient will be treated with radiotherapy in 3D conformal or IMRT/VMAT/Tomotherapy technique. Dose of radiotherapy to primary tumor, negative margin tumor bed, and close or positive margin tumor bed are 70, 60, and 66 Gy, respectively. Dose and area of neck node irradiation depend on physician judgement.

Chemotherapy

Platinum-based chemotherapy (cisplatin 40 mg/m^2 or carboplatin AUC 2) will be given in weekly basis during radiotherapy.

Induction chemotherapy or adjuvant chemotherapy is allowed. The induction/adjuvant chemotherapy regimen is based on each institutional practice.

Patient assessment

Basic characteristic data of patient

After patient signs informed consent form, the basic data including age, ECOG performance status, type of cancer and staging, plan of radiotherapy and chemotherapy will be collected.

Side effect evaluation

Radiotherapy acute side effect will be evaluated by CTCEA version 5[26] in weekly basis during radiotherapy in the following items.

- Oral mucositis
- Radiation dermatitis
- Esophagitis
- Laryngeal mucositis
- Pharyngeal mucositis
- Hematologic
    - Anemia
    - Decreased White blood cell
    - Decreased Neutrophil count
    - Decreased Lymphocyte count
    - Decreased Platelet count

Patients will also evaluate the oral mucositis and radiation dermatitis toxicities by themselves using Thai version of NCI- PRO-CTCAE[27].

Blood test

Complete blood count (CBC), BUN and creatinine (Cr) will be evaluated at 1-2 week before, and weekly during radiotherapy. Serum albumin will be collected at 1-2 week before and first, third and seventh week of radiotherapy.

ECOG performance status

ECOG performance status[28] will be assessed at 1 week before, weekly during radiotherapy, and 1 month after radiotherapy.

0	Fully active, able to carry on all pre-disease performance without restriction.
1	Restricted in physically strenuous activity but ambulatory and able to carry out work of a light or sedentary nature, e.g., light housework, office work.
2	Ambulatory and capable of all selfcare but unable to carry out any work activities. Up and about more than 50% of waking hours.
3	Capable of only limited selfcare, confined to bed or chair more than 50% of waking hours.
4	Completely disabled. Cannot carry on any selfcare. Totally confined to bed or chair.
5	Dead

Weight and Height

Weight and height will be measured weekly during radiotherapy. Body mass index (BMI) will be calculated by weight (kg) divided by square of height in centimeters.

$$\text{BMI} = \frac{\text{weight (kg)}}{[\text{height (cm)}]^2}$$

Nutritional status

All patients will receive nutrition consultation every visit.

Items	Screening	1-2 week before RT	Week during radiotherapy						
			1	2	3	4	5	6	7
Informed consent	x								
Demographic data	x								
Randomization	x								
CBC		x	x	x	x	x	x	x	x
BUN/Cr		x	x	x	x	x	x	x	x
Albumin		x	x			x			x

CTCEA		x	x	x	x	x	x	x	x
Weight		x	x	x	x	x	x	x	x
Height		x	x	x	x	x	x	x	x

Discontinuation/withdrawal of the volunteer from the study

The volunteer may with be withdrawn from the study anytime including:

- Patient withdraws consent (reason for withdrawal will be documented)
- Ineligibility (either arising during the study or retrospectively was overlooked at
- Screening)
- Significant non-compliance with treatment regimen or study requirements in inability to continue to comply with study procedures.
- An adverse event, which is deemed by the treating physician to compromise the safety of the subject, including grade III-IV toxicity caused by the study product.

Statistical Consideration

Study Endpoint

Primary endpoint

1. Difference of the proportion of grade 2-4 CTCEA toxicities of oral mucositis during radiotherapy between IN arm and control arm.

Secondary Outcomes

1. Difference of the proportion of remaining grade 2-4 CTCEA toxicities of each site/system of between 2 arms.
2. Difference of the proportion of remaining cumulative grade 2-4 PRO-CTCEA toxicities of oral mucositis and radiation dermatitis of between 2 arms.

Sample size

Based on our previous study on concurrent chemoradiotherapy (CCRT) in nasopharyngeal cancer, incidence of grade ≥2 oral mucositis according to CTCEA version 3 in CCRT and adjuvant chemotherapy was 61%.[29] The effect of IN on oral mucositis from a metaanalysis by Zheng et al.[20] showed that risk ratio of grade ≥3 was 0.45 (95% confident interval 0.22–0.92). We assume that IN will half the incidence of oral mucositis to 30.5%. With the power of 0.8 and beta of 0.05, the sample size is calculated to be 41 per arm. With 15% drop out rate is considered, the sample size per arm will be 49.

We will consider rounding up and including 50 participants per arm, the total participants in this study will be 100.

Statistical Analysis

The data will be presented in descriptive basis. The continuous data will be reported by using mean (or median) with standard deviation (or interquartile range). The dichotomous data will be reported using percentage or proportion. Comparing between arms will use student t-test or Wilcoxon Signed Rank Test with a significant level.

Interim Analysis

No interim analysis will be performed in this study.

Sponsor

Thai Otsuka Pharmaceutical Co, Ltd

Privacy of Patient Data

The collected data will be kept in the cabinet with the lock key or digitally kept in the computer that locks by the password. Only the investigators and regulatory authorities can assess the data.

Volunteer in the study will be coded by using the study-specific number. ID card number, hospital number, name, surname, or the other data that can be identified the identity of the volunteer will not be used or collected in the study.

The data will be retained in the study site for at least 2 years after the final analysis of the study according to ICH/GCH guideline.

Reference

1. Argirion I, Zarins KR, Defever K, Suwanrungruang K, Chang JT, Pongnikorn D, et al. Temporal changes in head and neck cancer incidence in Thailand suggest changing oropharyngeal epidemiology in the region. J Glob Oncol 2019;2019(5).
2. Tangjaturonrasme N, Vatanasapt P, Bychkov A. Epidemiology of head and neck cancer in Thailand. Asia Pac J Clin Oncol 2018;14(1):16–22.
3. Quon H, Vapiwala N, Forastiere A, Kennedy EB, Adelstein DJ, Boykin H, et al. Radiation therapy for oropharyngeal squamous cell carcinoma: American Society of Clinical Oncology endorsement of the American Society for Radiation Oncology evidence-based Clinical Practice guideline. J Clin Oncol 2017;35(36):4078–90.
4. Pfister DG, Spencer S, Adelstein D, Adkins D, Anzai Y, Brizel DM, et al. Head and neck cancers, version 2.2020. JNCCN J Natl Compr Cancer Netw 2020;18(7):873–98.
5. McLaughlin M, Patin EC, Pedersen M, Wilkins A, Dillon MT, Melcher AA, et al. Inflammatory microenvironment remodelling by tumour cells after radiotherapy. Nat Rev Cancer 2020;20(4):203–17.
6. Machon C, Thezenas S, Dupuy AM, Assenat E, Michel F, Mas E, et al. Immunonutrition before and during radiochemotherapy: Improvement of inflammatory parameters in head and neck cancer patients. Support Care Cancer 2012;20(12):3129–35.
7. Mendes RL, Nutting CM, Harrington KJ. Managing side effects of radiotherapy in head and neck cancer. Hosp Med2002;63(12):712–7.
8. Givens DJ, Karnell LH, Gupta AK, Clamon GH, Pagedar NA, Chang KE, et al. Adverse events associated with concurrent chemoradiation therapy in patients with head and neck cancer. Arch Otolaryngol - Head Neck Surg 2009;135(12):1209–17.
9. Alterio D, Marvaso G, Ferrari A, Volpe S, Orecchia R, Jereczek-Fossa BA. Modern radiotherapy for head and neck cancer. Semin Oncol2019;46(3):233–45.
10. Prieto I, Montemuiño S, Luna J, de Torres MV, Amaya E. The role of immunonutritional support in cancer treatment: Current evidence. Clin Nutr2017;36(6):1457–64.
11. Grimble RF. Basics in clinical nutrition: Immunonutrition – Nutrients which influence

immunity: Effect and mechanism of action. E Spen Eur E J Clin Nutr Metab 2009;4(1):e10–3.

12. McCowen KC, Bistrian BR. Immunonutrition: problematic or problem solving? Am J Clin Nutr 2003;77(4):764–70.

13. Cheng Y, Zhang J, Zhang L, Wu J, Zhan Z. Enteral immunonutrition versus enteral nutrition for gastric cancer patients undergoing a total gastrectomy: A systematic review and meta-analysis. BMC Gastroenterol 2018;18(1).

14. Xu J, Sun X, Xin Q, Cheng Y, Zhan Z, Zhang J, et al. Effect of immunonutrition on colorectal cancer patients undergoing surgery: a meta-analysis. Int J Colorectal Dis 2018;33(3):273–83.

15. Aeberhard C, Mayer C, Meyer S, Mueller SA, Schuetz P, Stanga Z, et al. Effect of preoperative immunonutrition on postoperative short-term outcomes of patients with head and neck squamous cell carcinoma. Head Neck 2018;40(5):1057–67.

16. Arends J, Bachmann P, Baracos V, Barthelemy N, Bertz H, Bozzetti F, et al. ESPEN guidelines on nutrition in cancer patients. Clin Nutr 2017;36(1):11–48.

17. Moya P, Soriano-Irigaray L, Ramirez JM, Garcea A, Blasco O, Blanco FJ, et al. Perioperative Standard Oral Nutrition Supplements Versus Immunonutrition in Patients Undergoing Colorectal Resection in an Enhanced Recovery (ERAS) Protocol. Medicine (Baltimore) 2016;95(21):e3704.

18. Kucuktulu E, Guner A, Kahraman I, Topbas M, Kucuktulu U. The protective effects of glutamine on radiation-induced diarrhea. Support Care Cancer 2013;21(4):1071–5.

19. Sunpaweravong S, Puttawibul P, Ruangsin S, Laohawiriyakamol S, Sunpaweravong P, Sangthawan D, et al. Randomized study of antiinflammatory and immune-modulatory effects of enteral immunonutrition during concurrent chemoradiotherapy for esophageal cancer. Nutr Cancer 2014;66(1):1–5.

20. Zheng X, Yu KKK, Wang G, Liu M, Li Y, Yu P, et al. Effects of Immunonutrition on Chemoradiotherapy Patients: A Systematic Review and Meta-Analysis. J Parenter Enter Nutr 2020;44(5):768–78.

21. Chitapanarux I, Pisprasert V, Tharavichitkul E, Jakrabhandu S, Klunklin P, Onchan W, et al. Randomized study of nutritional status and treatment toxicities of oral arginine, glutamine, and Omega-3 fatty acids during concurrent chemoradiotherapy for head and neck cancer patients. Funct Foods Heal Dis 2016;6(3):121–32.

22. Assenat E, Latournerie M, Thézenas S, Gaillet S, Janiszewski C, Flori N, et al. A prospective phase II study evaluating the efficacy of oral immune modulating formulae on acute oral mucositis during radiochemotherapy in head and neck neoplasms. E Spen Eur E J Clin Nutr Metab 2011;6:e171–7.

23. Boisselier P, Kaminsky MC, Thézenas S, Gallocher O, Lavau-Denes S, Garcia-Ramirez M, et al. A double-blind phase III trial of immunomodulating nutritional formula during adjuvant chemoradiotherapy in head and neck cancer patients: IMPATOX. Am J Clin Nutr 2020;112(6):1523–31.

24. Lyra M de MF, Meira JEC de, Guedes G da S, Bueno NB. Immunonutrition in head and neck cancer: Systematic review and metanalysis of its clinical and nutritional effects. Clin Nutr ESPEN 2021;41:30–41.

25. Chitapanarux I, Traisathit P, Chitapanarux T, Jiratrachu R, Chottaweesak P, Chakrabandhu S, et al. Arginine, glutamine, and fish oil supplementation in cancer

patients treated with concurrent chemoradiotherapy: A randomized control study. Curr Probl Cancer 2020;44(1).

26. National Cancer Institute. Common Terminology Criteria for Adverse Events (CTCAE) Common Terminology Criteria for Adverse Events (CTCAE) v5.0. 2017.

27. National Cancer Institute. NCI-PRO-CTCAE ITEMS-THAI-THAILAND Item Library Version 1.0 [Internet]. [cited 2021 Apr 29];Available from: https://healthcaredelivery.cancer.gov/pro-ctcae/

28. Oken M, Creech R, Tormey D, Horton J, Davis T, McFadden E, et al. Toxicity and response criteria of the Eastern Cooperative Oncology Group. Am J Clin Oncol 1982;5(6):7165009.

29. Chitapanarux I, Kittichest R, Tungkasamit T, Asakit T, Chomprasert K, Chakrabandhu S, et al. Two-year outcome of concurrent chemoradiation with carboplatin with or without adjuvant carboplatin/fluorouracil in nasopharyngeal cancer: A multicenter randomized trial. Curr Probl Cancer 2021;45(1).
